# Isovitexin Protects Mice from Methicillin-Resistant *Staphylococcus aureus*-Induced Pneumonia by Targeting Sortase A

**DOI:** 10.4014/jmb.2206.06007

**Published:** 2022-10-07

**Authors:** Lili Tian, Xinliang Wu, Hangqian Yu, Fengying Yang, Jian Sun, Tiezhong Zhou, Hong Jiang

**Affiliations:** 1 Institute of Animal Husbandry and Veterinary Medicine, Jinzhou Medical University, Jinzhou 121001, P.R. China; 2 Department of Pharmacy, Tianjin Baodi Hospital, Baodi Clinical College, Tianjin Medical University, Tianjin 301800, P.R. China; 3 College of Animal Science, Jilin University, Changchun 130062, P.R. China; 4 Department of Animal Husbandry and Veterinary Medicine, Beijing Vocational College Agriculture, Beijing 102442, P.R. China

**Keywords:** Methicillin-resistant *S. aureus*, anti-virulence, isovitexin, inhibitor, sortase A

## Abstract

The rise of methicillin-resistant *Staphylococcus aureus* (MRSA) has resulted in significant morbidity and mortality, and clinical treatment of MRSA infections has become extremely difficult. Sortase A (SrtA), a virulence determinant that anchors numerous virulence-related proteins to the cell wall, is a prime druggable target against *S. aureus* infection due to its crucial role in the pathogenicity of *S. aureus*. Here, we demonstrate that isovitexin, an active ingredient derived from a variety of traditional Chinese medicines, can reversibly inhibit SrtA activity in vitro with a low dose (IC_50_=24.72 μg/ml). Fluorescence quenching and molecular simulations proved the interaction between isovitexin and SrtA. Subsequent point mutation experiments further confirmed that the critical amino acid positions for SrtA binding to isovitexin were Ala-92, Ile-182, and Trp-197. In addition, isovitexin treatment dramatically reduced *S. aureus* invasion of A549 cells. This study shows that treatment with isovitexin could alleviate pathological injury and prolong the life span of mice in an *S. aureus* pneumonia model. According to our research, isovitexin represents a promising lead molecule for the creation of anti-*S. aureus* medicines or adjuncts.

## Introduction

*Staphylococcus aureus* is a commensal and opportunistic pathogen that causes a wide range of human diseases, from mild skin infections to more serious infections and even death [1]. In particular, the emergence and dissemination of methicillin-resistant *S. aureus* (MRSA) have been linked to significant morbidity and mortality, as well as the endangerment of human safety at great financial expense[2].

*S. aureus* employs a diverse set of virulence characteristics to produce a variety of illnesses while evading host immunological clearance [3]. These factors include surface-associated adhesins and secreted proteinaceous toxins [4]. The former interact with proteins of host tissues and cells and facilitate bacterial adherence to extracellular matrix components or internalization into cells [5, 6]. Proteinaceous toxins and enzymes released by bacteria may harm tissues and cells, aid bacteria in evading the human immune system, and accelerate the spread of bacteria in the host [7]. Small-molecule inhibitors that target *S. aureus* virulence are potential strategies because they disarm germs and enable the treatment of infections without subjecting them to selective pressure, which lowers the chances of antibiotic resistance.

Sortase A (SrtA) is an extensively studied, membrane-localized transpeptidase that is essential for the pathogenesis of *S. aureus* infections [8]. Strains with *srtA* gene mutations lack the cell wall-anchored proteins on the bacterial surface [9]. The inhibition of SrtA can significantly attenuate *S. aureus* virulence and improve the prognosis of abscesses, pneumonia, sepsis and infective endocarditis in various animal models of *S. aureus* infection [10-13]. Hence, SrtA is viewed as a desirable target for the creation of novel anti-MRSA drugs.

**Fig. 1:**
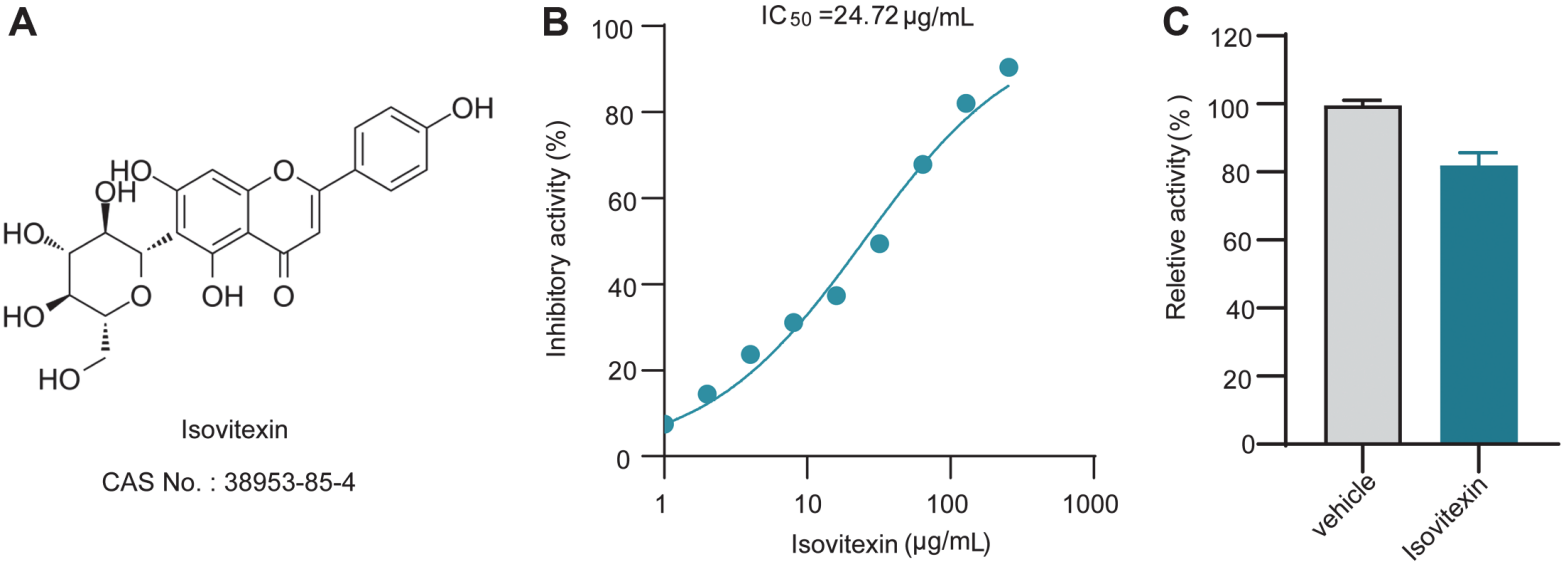
Isovitexin reversibly inhibits SrtA activity. (**A**) Chemical structure of isovitexin. (**B**) Isovitexin inhibits SrtA cleavage of the Abz-LPATG-Dap (Dnp)-NH_2_ substrate in a dose-dependent manner in vitro, and the activity of the untreated SrtA was set to 100%. (**C**) Isovitexin reversibly inhibits SrtA activity. SrtA was treated with or without 10 × IC_50_ of isovitexin and then diluted, and the activity of SrtA was determined by FRET assay. The activity obtained without SrtA (mock) was set to 100%.

There are several kinds of SrtA inhibitors that have been found to treat *S. aureus* infection in vivo, including natural plant products, small-molecule synthetic libraries, and peptidomimetics [14, 15]. Isovitexin (apigenin-6-C-glucoside, Fig. 1A) is a naturally occurring flavonoid of various edible plants, including the leaves of pigeon pea and *Ficus deltoidei* [16]. This compound possesses numerous pharmacological effects, including antioxidant, anti-inflammatory, and anticancer activities [17]. Our earlier research demonstrated that while isovitexin has no antibacterial effect against *S. aureus* in vitro, it prevents the growth of biofilms and SpA attachment to cell walls by repressing the activity of SrtA [18]. Moreover, though we found isovitexin to be a powerful SrtA inhibitor, the inhibitory mechanism and therapeutic effect against *S. aureus* infection in vivo remain unclear. In this study, after finding that isovitexin reversibly inhibited *S. aureus* SrtA, we assessed its therapeutic effect in vivo. Given the significance of SrtA in staphylococcal infection, our research indicates that isovitexin is a promising lead molecule whose potential as a treatment for MRSA infection warrants further study.

## Materials and Methods

### Bacterial Strains and Culture Conditions

MRSA USA300 used throughout this experiment was provided by American Type Culture Collection (ATCC, USA). The expression vector was built using *Escherichia coli* DH5α as a bacterial host. The bacteria were cultured in an incubator at 37°C with shaking at 220 ×*g*. For plasmid selection or upkeep, kanamycin (50 μg/ml) was occasionally added. Chengdu Rifenshi Co. (China) supplied isovitexin (purity > 98%). The HPLC quality test report for isovitexin has been added to Fig. 1 in the Supplementary Material.

### Preparation of Recombinant SrtA and Mutant Plasmids

The genomic DNA of *S. aureus* was amplified to produce the SrtA coding sequences. To construct pET28a-SrtA, the amplified segment was digested with NdeI and BamHI before being placed into the pET28a cloning site. Site-directed mutagenesis was performed using a mutagenesis kit and a recombinant vector of pET28a-SrtA. Table 1 lists each primer utilized in the study.

### SrtA and Mutant Proteins

For protein expression, plasmids were transformed into the expression host (TSC-E01, Tsingke Biotechnology Co., Ltd.). Then, 0.1 mM isopropylthio-ß-D-galactoside (IPTG) was used to induce the production of recombinant proteins overnight at 16°C. The tagged recombinant protein was purified using a His-tag purification resin (Beyotime, China) as previously reported [19].

### Measurement of SrtA Activity

The fluorescence resonance energy transfer (FRET) assay was used to assess SrtA activity [20, 21]. Briefly, the assay was carried out in 96-well, black plates with 10 μM SrtA and isovitexin for 30 min at 37°C. After that, a 10 M concentration of the SrtA substrate peptide Abz-LPATG-Dap (Dnp)-NH_2_ (LifeTein, China) was added and incubated for 30 min. As negative controls, wells containing all reagents but SrtA were employed. The wavelengths of emission and excitation were measured to be 395 and 420 nm, respectively.

### SrtA Reversible Inhibition Assay

A test for the reversible inhibition of SrtA was performed according to a previous report. Briefly, 200 μM of isovitexin was combined with SrtA (100 μl, 10 μM). Reaction buffer (9.9 ml) was added to the mixture after it had been incubating for 30 min. Then, 190 μl of the diluted mixture was combined with 10 μl (10 μM) of the substrate peptide. The fluorescence intensity was measured in the same way as in the SrtA activity assay.

### Internalization Assay

The details of the internalization assay are described in the Supplementary Materials.

### Fluorescence Quenching Analysis

The binding affinity of isovitexin to SrtA was further assessed using fluorescence quenching. Purified SrtA (5 μM) and different concentrations of isovitexin (0, 0.75, 4.5, 9, 13.5, 18 and 22.5 μg) were mixed in 1 mL of reaction buffer. Subsequently, the continuous wavelength fluorescence scanning instrument was used with excitation at 280 nm, a bandpass filter of 5 nm, and an emission slit width of 10 nm. The fluorescence emission spectra of the mixed solutions were then measured (260–400 nm). Details of the experiments and the calculation of *K_A_* values were reported previously [22].

### Western Blot Analysis

The details of the western blot analysis are described in the Supplementary Material.

### Molecular Modeling

Molecular docking was performed using the AutoDock Vina 1.1.2 package on the basis of the isovitexin and SrtA protein structures (PDBID:1T2P) [23]. Using the Amber14 software program, the best docked position (conformation) in the isovitexin-SrtA complex was put through molecular dynamics simulations (25 ns) [24-26], which were performed as described previously [27].

### Acute Toxicity Assay and Mouse Model of *S. aureus* Pneumonia

The details of the acute toxicity assay and mouse model of *S. aureus* pneumonia are described in the Supplementary Material.

### Statistical Analysis

The experimental data for each group in the individual experiments are expressed as the means ± SD, and were analyzed using GraphPad Prism 8.0. *p* < 0.05 was used to denote statistical significance.

## Results

### Isovitexin Reversibly Inhibits SrtA Activity

SrtA activity was inhibited in a dose-dependent manner by isovitexin (Fig. 1B). Subsequently, we further determined whether isovitexin’s inhibition of SrtA is reversible. The activity of SrtA showed 83 ± 2.31% recovery in comparison with the vehicle group (Fig. 1C), which demonstrated that the suppression of SrtA by isovitexin was reversible and that isovitexin interacted noncovalently with SrtA.

### Isovitexin Suppresses the Internalization of *S. aureus* into A549 Cells

**Fig. 2:**
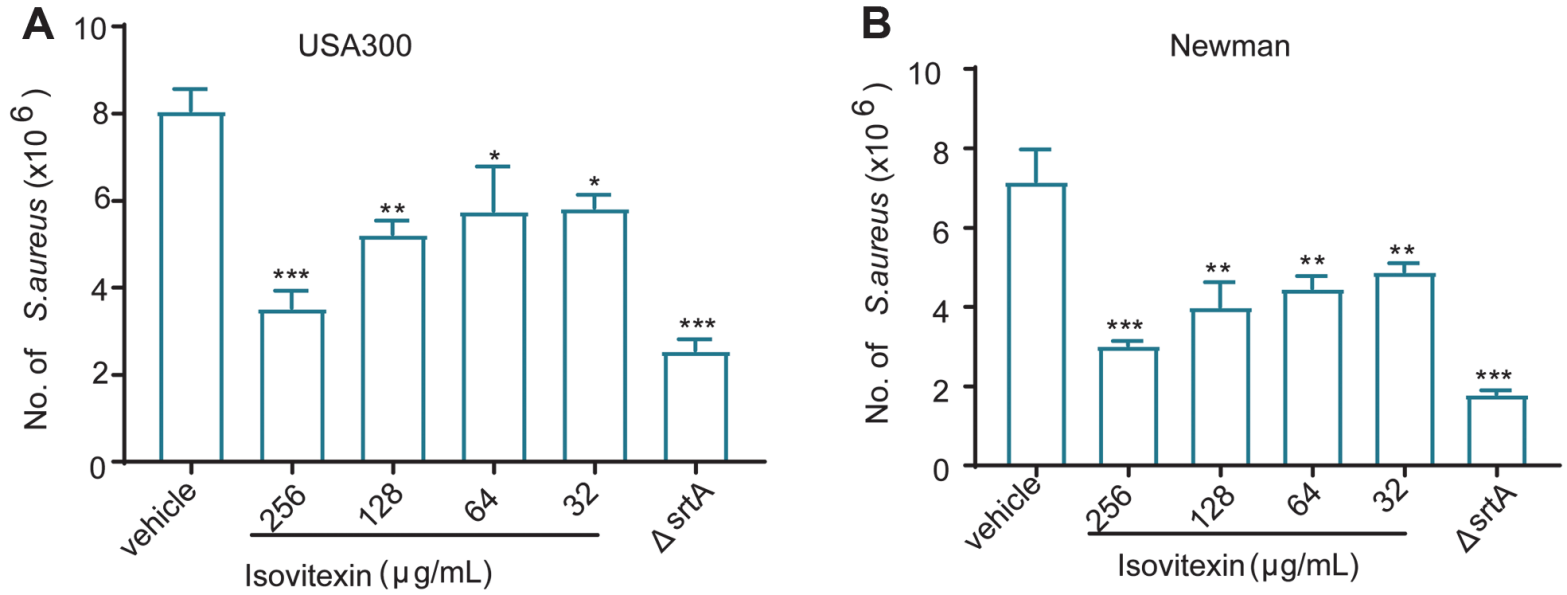
Isovitexin affects the internalization of *S. aureus* into A549 cells. (**A**) A549 cells were infected with *S. aureus* USA300 or (**B**) *S. aureus* Newman pretreated with different concentrations of isovitexin and lysed 2 h postinfection, and the number of viable *S. aureus* in the cells was quantified by serial dilution on LB agar plates. The error bars indicate the mean ± SD of three replicates. *p < 0.05, **p < 0.01, and ***p < 0.001 calculated by two-tailed Student’s *t*-test.

We then used the epithelial cell line A549 to determine whether isovitexin could block *S. aureus* internalization into the lung epithelial cells. As shown in Fig. 2A, 256 μg/ml isovitexin treatment significantly decreased the amount intracellular *S. aureus* USA300 compared to the DMSO-treated control (vehicle). Similar results were also observed with the *S. aureus* Newman group (Fig. 2B). In summary, these findings suggest that suppressing SrtA with isovitexin limits the penetration of *S. aureus* into epithelial cells.

### Isovitexin Does Not Inhibit the Expression of SrtA

**Fig. 3:**
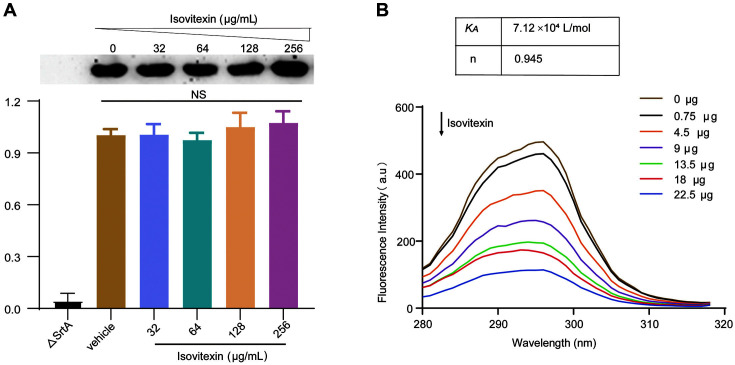
Expression level of SrtA and interaction between isovitexin and SrtA measured by a fluorescence quenching assay. (**A**) Western blot analysis of SrtA protein from *S. aureus* treated with various concentrations of isovitexin (0 to 256 μg/ml). (**B**) A fluorescence quenching assay was performed to assess the binding affinity of isovitexin to SrtA, and a *K_A_* value of 7.12 × 10^4^ l/mol was calculated. n represents the number of binding sites.

*S. aureus* was treated with isovitexin at various concentrations (0-256 μg/ml) to determine if it suppressed SrtA expression. Isovitexin therapy had no effect on SrtA expression, as indicated in Fig. 3A. The result indicated that the suppressive effect was not achieved by isovitexin affecting the expression of SrtA.

### Strong Affinity Between Isovitexin and SrtA

The binding capacities of isovitexin and SrtA were evaluated using fluorescence quenching test based on the premise of quenching fluorescence signals upon target binding. This method can also assess the binding capacity, binding characteristics, and kinetically relevant properties between the drug and the protein. The fluorescence intensity of the SrtA protein decreased significantly with increasing isovitexin concentrations, as illustrated in Fig. 3B. Because the binding of WYBQ-4 to the PBP2a protein is static quenching, the binding constant *K_A_* can be calculated according to the static quenching formula Lg[(F_0_-F)/F] = Lg *K_A_* + nlg Q. The *K_A_* value of 7.12 × 10^4^ l/mol was further calculated, which confirmed a strong affinity between isovitexin and SrtA.

### Binding Mode of Isovitexin with SrtA

**Fig. 4:**
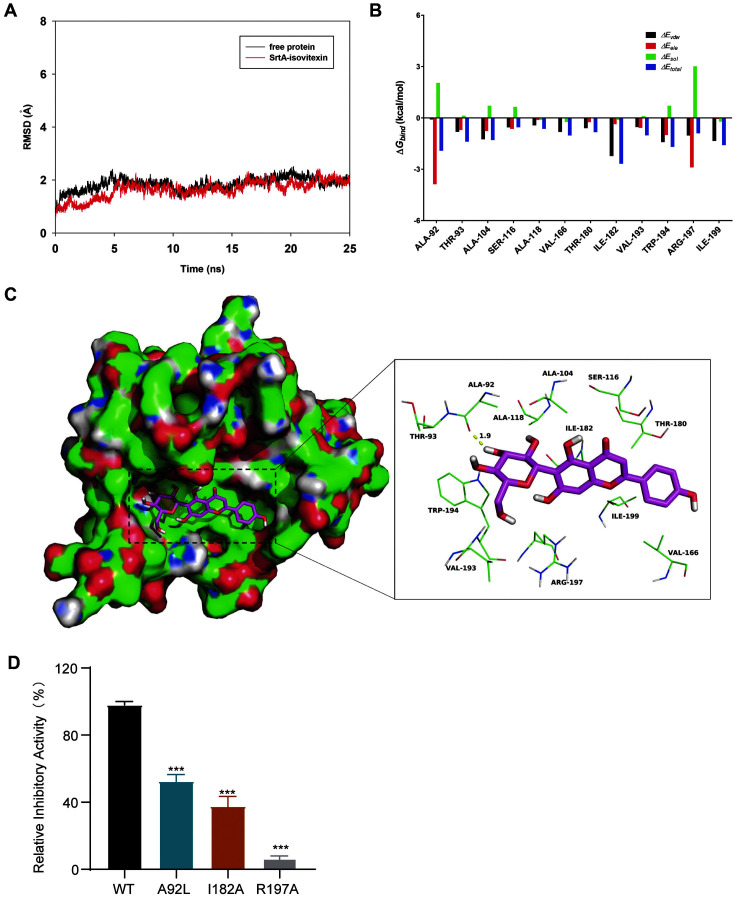
Molecular modeling of the interaction between isovitexin and SrtA. (**A**) RMSD (Å) of the modeled *S. aureus* SrtA backbone based on the initial structure during MD simulation. (**B**) Decomposition of the binding free energy on a perresidue basis between isovitexin and modeled *S. aureus* SrtA. (**C**) Docking model of isovitexin within SrtA during MD simulation. (**D**) FRET was used to evaluate the transpeptidase activity of isovitexin (64 μg/ml) on SrtA and mutant proteins.

Molecular dynamics simulations were conducted to elucidate the mechanism through which isovitexin inhibits SrtA. To determine the dynamic stability of the docking model and the rationality of the method, the root-mean-square deviation (RMSD) value over time for the SrtA backbone in the whole complex was calculated and plotted. The results showed that the protein structure of the system achieved a steady state (Fig. 4A). As is shown in Fig. 4C, the results indicate that van der Waals interactions, electrostatic, and hydrogen bonding appear to be the main forces involved in the interactions between isovitexin and SrtA. In detail, residue Ala-92 is proximal to the glucose group of isovitexin and forms a hydrogen bonding interaction (bond length: 1.9 Å) between SrtA and isovitexin (Fig. 4C). The MM-GBSA approach was used to determine the total contribution of residues at the isovitexin binding site to the binding free energy. In the SrtA-isovitexin complex, residue Ala-92 has a favorable electrostatic (E_ele_) contribution with ΔE_ele_ <– 3.5 kcal/mol (Fig. 4B). In addition, residue Arg-197 is close to the flavone scaffold of isovitexin, forms a cation-π interaction, and exhibits a moderate ΔE_ele_ contribution with ΔE_ele_ <– 2.5 kcal/mol (Fig. 4B). Moreover, the side chains of Ala-104, Ile-182, Trp-194, and Ile-199 with ΔE_vdw_ <– 1.0 kcal/mol form strong van der Waals interactions with isovitexin because these residues are close to isovitexin. Moreover, van der Waals contacts accounted for the bulk of the decomposed energy interactions, primarily through hydrophobic interactions like those found for Ala-92, Ala-104, Ala-118, Val-166, Ile-182, Val-193, Trp-194, and Ile-199. Additionally, the total binding free energy for the SrtA-isovitexin complex was calculated, and an estimated ΔG_bind_ of –15.7 kcal/mol was found for isovitexin, which suggested that isovitexin can bind to SrtA robustly.

Given the outcomes of the molecular dynamics simulation of isovitexin's binding to SrtA, we first mutated the key amino acids Ala-92, Ile-182, and Arg-197. The relative transpeptidase activity of isovitexin (64 μg/ml) on SrtA and its mutant proteins was also determined. As illustrated in Fig. 4D, the transpeptidase inhibitory activity of isovitexin on mutated SrtA was significantly reduced to different degrees compared with that found for the WT group, which indicated that Ala-92, Ala-182, and Arg-197 were identified as necessary sites for binding. In summary, the above-described molecular modeling provides support for the interaction between isovitexin and SrtA.

### Isovitexin Protects Mice from *S. aureus* Pneumonia

Since isovitexin inhibited SrtA activity and suppressed the invasion of *S. aureus* into the epithelial cell line A549, we subsequently sought to determine whether isovitexin exerts protective effects in vivo. Before conducting formal animal experiments, the acute toxicity was assessed by administering 50-200 mg/kg isovitexin intraperitoneally, and none of the groups of mice showed discomfort or delayed or inert behavior. Even a single administration of 200 mg/kg isovitexin is safe for mice (Fig. S2).

**Fig. 5:**
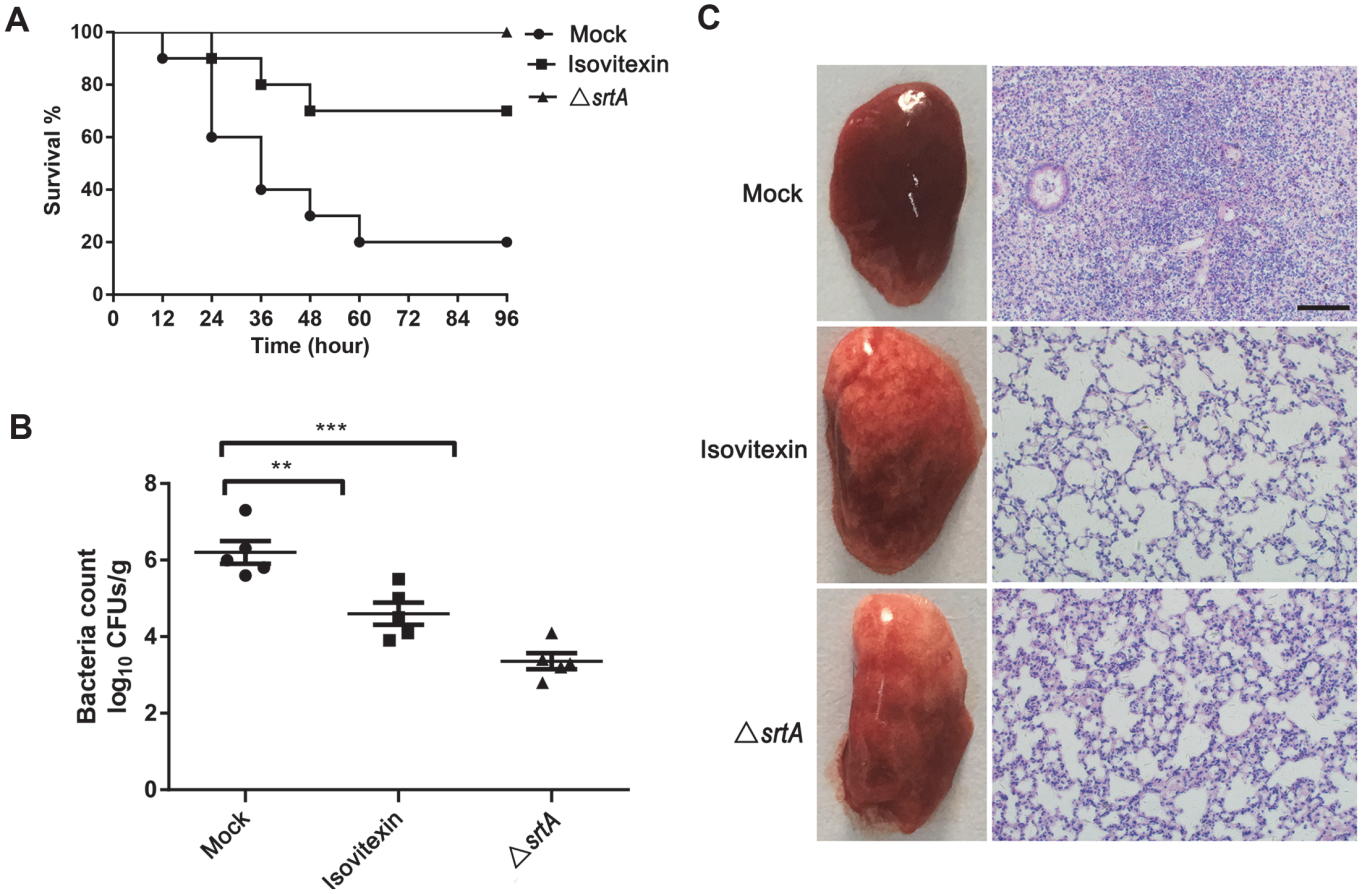
Effect of isovitexin treatment on *S. aureus*-induced pneumonia in mice. (**A**) Effect of isovitexin treatment on the survival of mice (*n* = 10) infected with a lethal dose of *S. aureus*. Mock vs. isovitexin, **p < 0.01; log-rank test. (**B**) Effect of isovitexin treatment (100 mg/kg) on the bacterial load in lungs of mice (*n* = 5). **p < 0.01, ***p < 0.001; Mann‒Whitney test, twotailed. The horizontal bars represent the means. (**C**) Histopathology of lungs (H&E-stained tissues) of mice treated or not treated with isovitexin (100 mg/kg). The animal data were obtained from two separate experiments.

Following intranasal inoculation (i.n.) of 7-week-old mice with *S. aureus* and treatment with isovitexin every 12 h, the percent mortality of the isovitexin-treated group at 24, 48, and 72 h was significantly lower than that of the control group (p = 0.005, 0.032 and 0.025, respectively) (Fig. 5A). Furthermore, compared to the mock-treated group, isovitexin treatment decreased the amount of live *S. aureus* in lung tissues (Fig. 5B).

A gross examination of lung tissues was first performed and revealed that the lung tissues treated with isovitexin were pink and spongy, while the untreated infected micés lung tissues were superficially mottled, red, and contained numerous focal infections (Fig. 5C, left panel). Histopathologic analysis revealed that inflammatory cells infiltrated the majority of the alveolar spaces in the mock group, and that treatment with isovitexin significantly alleviated inflammation, as evidenced by a reduction in the accumulation of inflammatory cells in the alveolar spaces (Fig. 5C, right panel). Taken together, the results indicate that isovitexin can attenuate *S. aureus* virulence and provide protection in vivo.

## Discussion

*S. aureus* is a leading bacterial cause of hospital- and community-acquired pneumonia. The proportion of MRSA isolated from infectious pneumonia patients has gradually increased in recent years, resulting in significant morbidity and mortality. Hence, the creation of new and potent anti-infective strategies is urgently needed to control MRSA infections. Due to the clear link between virulence mechanisms and pathogenesis, many studies have investigated antivirulence strategies.

SrtA has been identified as a prospective pharmaceutical target for antivirulence therapy because it mediates proteins anchoring to the cell surface of bacteria [28]. These surface proteins have been linked to bacterial colonization of host tissues and cells, the development of biofilms, and immune response evasion. Therefore, targeting SrtA and blocking the anchoring of surface proteins is a promising therapeutic approach to cope with *S. aureus* infection.

Natural compounds have long been recognized for their safety and environmental friendliness, making them appealing in the fight against diseases [29]. During the sorting signal reaction in *S. aureus*, SrtA was recognized to cleave the LPXTG motif between the threonine and glycine residues in order to covalently attach the protein to the cell surface of bacteria via a transpeptide reaction. Therefore, the FRET method based on this principle has become the current weapon of choice for screening SrtA inhibitors. Herein, we identified the natural product isovitexin and found that it substantially reduces SrtA activity, with an IC_50_ value of 24.72 μg/ml. Subsequently, we also demonstrated that isovitexin inhibits SrtA in a reversible manner. This finding is encouraging because isovitexin is a member of the flavonoid family and has no known biohazards.

Strains with *srtA* mutation display diminished virulence in multiple animal models [30], indicating that SrtA is crucial in the pathogenesis of staphylococcal infection. Therefore, inhibiting the anchoring of surface proteins by interfering with SrtA activity will affect *S. aureus* and thus its establishment of pulmonary infection. As expected, isovitexin dramatically decreased *S. aureus*'s internalization ability in A549 cells. The mechanism of decreased *S. aureus* internalization by the inhibition of SrtA may be attributed to reductions of cell-surface proteins.

A fluorescence quenching assay confirmed that isovitexin directly inhibited SrtA. Molecular modeling of the interaction of isovitexin with SrtA was subsequently conducted, and the results revealed that isovitexin’s interaction with the binding pocket of SrtA was primarily dependent on van der Waals interactions, and electrostatic and hydrogen bonding. Additionally, treatment with isovitexin markedly improved the survival rate of mice infected with a lethal dose of MRSA, decreased the survival of bacteria in lung tissues, improved the pathological injury of lung tissues, and attenuated pulmonary inflammation. These therapeutic effects were probably due to the reduction in bacterial adhesion and invasion of epithelial cells because of SrtA inhibition. These data demonstrated that isovitexin is effective for treating *S. aureus* pneumonia.

Furthermore, using the SwissADME website (http://www.swissadme.ch/index.php), we confirmed that isovitexin does not contain a PAINS moiety. This finding in combination with our experimental results confirmed that isovitexin is specific for binding to SrtA. Moreover, isovitexin reportedly inhibits coagulase, another important virulence factor of *S. aureus* [31]. When *S. aureus* invades the body, the secreted coagulase can bind to thrombinogen in blood or plasma, and thus converts fibrinogen into fibrin to wrap around the surface of the bacterium. The formed fibrin network can then protect *S. aureus* from phagocytosis and promotes platelet aggregation and microthrombosis, which leads to staphylococcal abscess lesions and fatal bacteremia. Isovitexin acts as a multiple *S. aureus* virulence inhibitor, which implies a more significant anti-infective effect compared with that obtained with a single virulence target. To sum up, isovitexin is a promising lead molecule for additional research into the creation of an antivirulence medication for the treatment of MRSA infections.

## Ethics Approval

The animal work was approved by the Institutional Animal Care and Use Committee (IACUC) of Jilin University and followed all standards regarding the use of experimental animals. (Permit No. SY202110100).

## Figures and Tables

**Table 1:** Primers used in this study.

Primer name	Sequences (5’-3’)
srtA-F	GGGAATTCCATATGCAAGCTAAACCTCAAATTCCG
srtA-R	CGCGGATCCTTATTTGACTTCTGTAGCTACAAAGA
A92L-srtA-F	GACCAAAAACACCTGAACAATTAAA
A92L-srtA-R	CTGGATATACTGGTTCTTTAATATCAGC
I182A-srtA-F	AACATTAGCTACTTGTGATGATTAC
I182A-srtA-R	AATTGTTTATCTTTACCTTTTTGTTCA
R197A-srtA-F	GGAAAAAGCTAAAATCTTTGTAGCT
R197A-srtA-R	CAAACGCCTGTCTTTTCATTG
